# Efficient adsorption of cesium cations and chromate anions by one-step process using surfactant-modified zeolite

**DOI:** 10.1007/s11356-023-25644-y

**Published:** 2023-02-28

**Authors:** Moustafa A. Hamoud, Shereen F. Abo-Zahra, Mohamed A. Attia, Hanan H. Someda, Mamdoh R. Mahmoud

**Affiliations:** Nuclear Chemistry Department, Radioisotopes Production and Radiation Sources Division, Hot Laboratories Center, Egyptian Atomic Energy Authority, P.O. Box 13759, Cairo, Egypt

**Keywords:** Cesium, Chromium, Adsorption, Zeolite, Surfactant

## Abstract

Natural zeolite is organically modified with the surfactant cetyltrimethylammonium bromide (CTAB) and employed as a dual-function material for simultaneous adsorption of Cs^+^ cations and HCrO_4_^−^ anions from aqueous solutions. Unmodified and modified zeolites are characterized by Fourier transform infrared (FTIR), dynamic light scattering (DLS), nitrogen adsorption–desorption isotherms, and X-ray diffraction (XRD). The results showed that CTAB-zeolite had the efficiency to simultaneously adsorb the concerned species in the pH range 2.5–4.2. The kinetic data showed that 90 and 300 min for Cs(I) and Cr(VI), respectively, were sufficient to attain equilibrium and the data are well-fitted by the double-exponential kinetic model. Of the studied adsorption isotherm models, Redlich-Peterson was the best one for describing the equilibrium adsorption isotherms. Values of ∆*H*°, ∆*S*°, and ∆*G*° for the present adsorption processes are estimated. CTAB-zeolite exhibited adsorption capacities of 0.713 and 1.216 mmol/g for Cs(I) and Cr(VI), respectively, which are comparable with the data reported in the literature. The adsorption mechanism of the concerned (radio)toxicants is proposed.

## Introduction

The high water solubility and mobility, long half-life, high specific radioactivity, and comparable ionic radius (2.44 A°) to that of potassium (0.280 nm) make radioactive cesium to be one of the most hazardous and problematic radionuclides (Park et al. 2021; Kim et al. 2020). The other contaminant, hexavalent chromium (Cr(VI)), exists in the aquatic environment in various anionic forms of HCrO_4_^−^, Cr_2_O_7_^2−^, and CrO_4_^2−^ depending on the pH of the medium (Zekavat et al. 2020).Radionuclides of cesium and chromium co-exist in radioactive liquid wastes generated from radioisotope production facilities and/or radiochemistry research laboratories. Among the various technologies applied for the removal of (radio)contaminants from liquid wastes which include solvent extraction, chemical precipitation, reverse osmosis, membrane separation, and adsorption (Kim et al. 2021; Pineda et al. 2021; Chen et al. 2020; Rogers et al. 2012), the last one is often preferred due to its high efficiency, simplicity, low-cost, and reversibility. Inorganic, organic, and composite materials are the three known types of adsorbents used for this process. Because of their unique advantages of low cost, availability, and large quantities (the world’s annual production of natural zeolite in 2016 was approximately 3 million tons), and high adsorption capacity as well as their good mechanical, thermal and radiological stabilities, zeolites are considered the promising inorganic adsorbents for removal of (radio)toxicants from water systems (Rad and Anbia 2021; Montalvo et al. 2020; Kim et al. 2020; Wang et al. 2018).

Natural zeolites, hydrated aluminosilicate porous materials, are cage-like structures consisting of three-dimensional frameworks of SiO_4_ and AlO_4_ tetrahedral. The isomorphous substitution of tetravalent silicon (Si^4+^) with trivalent aluminum (Al^3+^) produces a negative charge in the lattice which is compensated by exchangeable cations such as K^+^, Na^+^, Ca^2+^, and Mg^2+^. These non-toxic cations are weakly held and can be exchangeable with other cations in solutions (Abdollahi et al. 2020). Accordingly, natural zeolites possess a negatively charged surface and exhibit cation-exchange properties (Szala et al. 2015; Warchol et al. 2006), while have no ability to adsorb anions. To enhance their adsorption efficiency toward anionic species, natural zeolites have been modified to convert the surface charge from negative to positive. Cationic surfactants were extensively used for this purpose where they are characterized by their strong binding with negatively charged surfaces (Haggerty and Bowman 1994; Boyd et al. 1988). Adsorption of cationic surfactants, such as cetyltrimethylammonium bromide (CTAB), onto negatively charged surfaces has been kinetically and thermodynamically studied (Li 1999; Sullivan et al. 1998) and it governs via two stages depending on the surfactant concentration. Below its critical micelle concentration (cmc), the cationic surfactant is adsorbed at the solid–liquid interface by electrostatic attraction with the formation of a monolayer or “hemimicelle.” At concentrations higher than the cmc, the long hydrocarbon chains of the surfactant molecules associate by the hydrophobic effect to form bilayer or “admicelle.” This arrangement resulted in the formation of a positively charged surface as the polar groups of the surfactant, which carry a positive charge, are directed to the liquid phase. Therefore, cationic surfactant-modified zeolites are known for their ability to adsorb anionic contaminants from aqueous solutions via anion exchange with the counter ion of the surfactant (Nasanjargal et al. 2021; Szala et al. 2015; Hommaid and Hamdo 2014; Swarnkar and Radha 2011; Zeng et al. 2010; Warchol et al. 2006; Ghiaci et al. 2004; Haggerty and Bowman 1994). In reviewing these previous publications, it is found that the researchers are mainly concerned with the adsorption of anionic contaminants only and few studies are reported on simultaneous adsorption of cationic and anionic species. Chao and Chen modified NaY zeolite with hexadecyltrimethylammonium bromide (HDTMA) for adsorption of cationic (Cu^2+^, Zn^2+^, Ni^2+^, Pb^2+^, and Cd^2+^) and oxyanionic (Cr_2_O_7_^2−^ and MnO_4_^−^) metal ions with maximum adsorption capacities of 0.388, 0.318, 0.315, 0.653, 0.351, 0.184, and 0.412 mmol/g, respectively (Chao and Chen 2012). Removal of Cu^2+^, Zn^2+^, Ni^2+^, Pb^2+^ and Cd^2+^, Cr_2_O_7_^2−^, and MnO_4_^−^ heavy metals from water by adsorption onto HDTMA-modified zeolite is investigated by Huang et al. (2016). Zekavat et al. (2020) used HDTMA-modified zeolite for the simultaneous adsorption of copper cations (Cu^2+^) and hexavalent chromium anions (HCrO_4_^−^ and Cr_2_O_7_^2−^) from aqueous solutions. Maximum adsorption capacities of 0.068 and 0.0093 mmol/g are achieved for Cu^2+^ and Cr(VI), respectively. As shown, none of these publications have studied the simultaneous adsorption of ^134^Cs^+^ and HCrO_4_^−^ onto surfactant-modified zeolite, which is the main objective of the current study.

## Experimental

### Materials and reagents

Natural zeolite used in this study was purchased from A&O trading company, Egypt. The cationic surfactant cetyltrimethylammonium bromide (CTAB, purity > 98%) was supplied by Merck and was used for the modification of natural zeolite. Stock solutions of Cs(I) and Cr(VI), 0.5 mol/L of each, were prepared by dissolving the appropriate amounts of cesium chloride (CsCl, 99.99% purity, Sigma) and potassium chromate (K_2_CrO_4_, 99% Purity, Aldrich) in distilled water, respectively. The radioactive Cs-134, obtained by dissolving the irradiated CsCl in the Egypt Second Research Reactor in distilled water, was used as a radiotracer for radio-analysis of cesium during adsorption experiments. Potassium chloride (KCl), ammonium sulfate ((NH_4_)_2_SO_4_), and aluminum nitrate (Al(NO_3_)_3_) were supplied by Riedel-de-Haen, while sodium carbonate (Na_2_(CO_3_)) and calcium nitrate (Ca(NO_3_)_2_) were obtained from Chem-Lab. These salts were used for studying the effect of foreign ions on the adsorption efficiency of the concerned (radio)toxicants onto modified zeolite. Sodium hydroxide (NaOH) and hydrochloric acid (HCl) were purchased from Chem-Lab and were utilized for adjusting the solution pH.

### Modification of natural zeolite

Modification of natural zeolite was governed by adding 10 g of natural zeolite to 100 mL of CTAB solution of different concentrations (0, 0.01, 0.05, 0.075, 0.1 mol/L). The suspensions were magnetically stirred at 200 rpm for 24 h at 25 °C. The solid phase was then separated by decantation, thoroughly washed with distilled water, and finally dried at 70 °C until constant weight. The produced material, denoted CTAB-Zeolite, was kept in a closed bottle for subsequent use.

### Characterization

The Fourier transform infrared (FTIR) spectra of natural zeolite and CTAB-zeolite were obtained by a Nicolet iS10-FTIR spectrometer (USA) in the range 4000–400 cm^−1^ at a resolution of 4 cm^−1^ using the KBr pellet method. The intensity-based particle size distribution for natural zeolite and CTAB-zeolite were studied through dynamic light scattering (DLS) measurements using a Malvern Zetasizer Nano instrument (Worcestershire, UK). DLS experiments were performed with a 4 mV He–Ne laser (wavelength = 633 nm) at 25 °C. The specific surface area, pore volume, and pore size of natural zeolite before and after the modification process with cetyltrimethylammonium bromide were estimated by nitrogen adsorption–desorption measurements using a NOVA Station A (Quantachrome instruments, USA). The X-ray diffraction patterns of zeolite before and after loading with cetyltrimethylammonium bromide surfactant were recorded using a Philips PW1830 diffractometer with Cu Kα as the incident radiation.

### Adsorption experiments

Adsorption experiments of this study were conducted by batch-type equilibration method in 25-mL glass bottles using a thermostated water bath shaker (Karl Kolb type, D-6072, Driesch, Germany). Unless otherwise stated, the working solutions had 0.25 mmol/L Cs(I) spiked with Cs-134 and 0.5 mmol/L Cr(VI) and the operating conditions which includes the adsorbent mass, pH of the solution, contact time, and temperature were kept constant at 6 g/L, 3.2, 24 h, and 25 °C, respectively. The influence of CTAB concentration on the adsorption efficiency of Cs(I) and Cr(VI) ions was investigated by contacting 6 g/L of zeolite functionalized at different concentrations of the surfactant (0, 0.01, 0.05, 0.075, and 0.1 mol/L) with the adsorbates (cesium and hexavalent chromium) solution for 24 h. The effect of the solution pH in the range 2.5–11.2 was studied by adding 0.03 g of the adsorbent (either natural zeolite or CTAB-zeolite) to 5 mL solution of the concerned (radio)toxicants adjusted to the required pH value using a HANNA pH-meter (model HI 8519, Italy). The samples were shaken for 24 h which were found from preliminary experiments to be more sufficient than required to attain equilibrium. Adsorption kinetics were investigated by mixing 5 mL of adsorbates solution adjusted to pH 3.2 with 0.05 g CTAB-zeolite for different time intervals. Adsorption isotherms were obtained by dispersing 0.03 g CTAB-zeolite in 5 mL of the working solutions, adjusted at pH 3.2, of different concentrations of Cs(I) and Cr(VI) in the range 0.05–10 mmol/L. To determine the appropriate adsorbent mass required to achieve efficient adsorption for Cs(I) and Cr(VI) ions, the organo-modified zeolite of different masses (0.005–0.05 g) was added to the (radio)toxicants solution of pH 3.2 and the samples were equilibrated for 24 h. The impact of temperature was studied by contacting 5 mL adsorbates solutions of Cs(I) and Cr(VI) in their binary systems to 0.05 g CTAB-zeolite at varying temperatures in the range 30–60 °C.

For estimating the maximum adsorption capacities of natural zeolite and CTAB-zeolite for cesium and hexavalent chromium, 0.1 g of the adsorbent were contacted with 10 mL of adsorbates solution (0.1 mmol/L of each, pH 3) for 24 h at 25 °C. The solid phases were separated, after equilibration, by centrifugation for measurement radiometric and spectrophotometric analysis of Cs-134 and Cr(VI) in the aqueous phase, respectively. Based on these measurements, the adsorbed amounts were calculated. Loading of these (radio)toxicants onto natural zeolite and CTAB-zeolite was repeated at the aforementioned optimum conditions until saturation of the adsorbents was attained.

### Analysis and data presentation

After equilibration, the solid phase was separated by centrifugation at 4000 rpm using a Chirana centrifuge (Germany). The gamma radioactivity of Cs-134 in the aqueous phase was measured radiometrically using a NaI scintillation detector connected to a Specteh ST360 single-channel spectrometer (USA). On the other hand, chromium concentration was determined spectrophotometrically using a Spectronic-20 Uv–vis spectrophotometer (USA). Thereafter, the data obtained were presented as adsorption percentage (adsorption %), adsorbed amount (*q*), and distribution coefficient (*K*_d_) which were calculated using the following relations:

1 $$\mathrm{Adsorption\;\% \;of\;Cs}-134=\left[1-\frac{{A}_{\mathrm{initial}}}{{A}_{\mathrm{final}}}\right]\times 100$$

2 $$\mathrm{Adsorption\;\%\;of\;Cr}\left(\mathrm{VI}\right)=\left[1-\frac{{C}_{\mathrm{initial}}}{{C}_{\mathrm{final}}}\right]\times 100$$

3 $$\mathrm{Adsorbed\;amount}=\frac{\mathrm{adsorption\;\%}\times {C}_{\mathrm{initial}}\times\;V}{100\times M}$$

4 $${K}_{\mathrm{d}}{(\mathrm{L}/\mathrm{g})}_{\mathrm{Cs}-134}=\left[\frac{{A}_{\mathrm{initial}}}{{A}_{\mathrm{final}}}-1\right]\times \left[\frac{V}{M}\right]$$

5 $${K}_{d}{(L/g)}_{\mathrm{chromium}}=\left[\frac{{C}_{\mathrm{initial}}}{{C}_{\mathrm{final}}}-1\right]\times \left[\frac{V}{M}\right]$$

### Error functions

Two commonly used error functions, namely, residual sum of square (RSS) and chi-square (*χ*^2^) are used during the modeling of kinetic and isotherm data of Cs(I) and Cr(VI) onto CTAB-zeolite to find out the most appropriate model to represent the experimental data. These error functions are expressed as (Mahmoud et al. 2014; Hararah et al. 2012):

6 $$\mathrm{RSS}=\sum_{i=1}^{{N}_{\mathrm{d}}}{\left(\mathrm{Experimental\;}{q}_{\mathrm{e}}\mathrm{\;value}-\mathrm{Predicted\;}{q}_{\mathrm{e}}\mathrm{\;value}\right)}^{2}$$

7 $${\mathrm{x}}^{2}=\sum_{\mathrm{i}=1}^{{\mathrm{N}}_{\mathrm{d}}}\frac{{\left(\mathrm{Experimental\;}{q}_{\mathrm{e}}\mathrm{\;value}-\mathrm{Predicted\;}{q}_{\mathrm{e}}\mathrm{\;value}\right)}^{2}}{\mathrm{Predicted\;}{q}_{\mathrm{e}}\mathrm{\;value}}$$

where *q*_e_ is the amount of adsorbate loaded onto CTAB-zeolite at equilibrium (mmol/g) and *N*_d_ is the number of experimental data points. The lower the RSS and *χ*^2^ values the better the ability of the model to fit the experimental data (Sun and Byrne 1998). Kinetic and isotherm parameters of the models studied in the present investigation were obtained by non-linear regression analysis method using OriginPro 8.5 software.

## Results and discussion

### Characterization

#### Fourier transform infrared

**Fig. 1:**
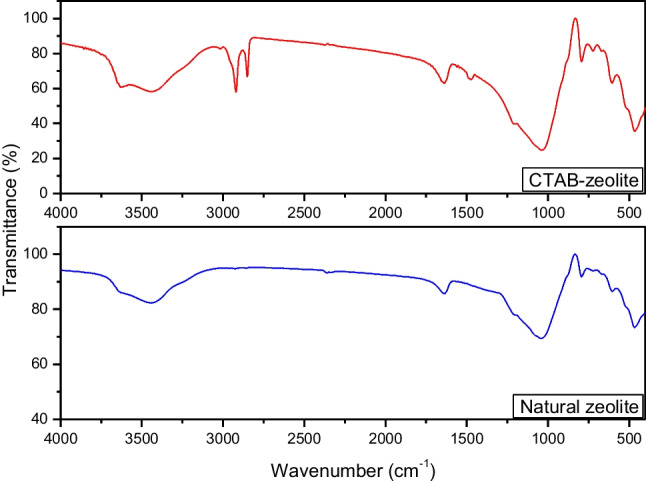
FT-IR spectra of natural zeolite and CTAB-zeolite

The FTIR spectra of natural zeolite and surfactant-modified zeolite are shown in Fig. 1. It can be noticed that both of the compared materials exhibited absorption peaks at 3628, 3425, 1642, 1032, 789, and 468 cm^−1^. The peak at 3628 cm^−1^ corresponds to the stretching vibration of Si–OH, whereas the broad band centered at 3425 cm^−1^ and that at 1642 cm^−1^ are corresponding to water molecules associated with exchangeable cations in zeolite structure (Nasanjargal et al. 2021; Wang et al. 2018). The peak at 1032 cm^−1^ is assigned to the asymmetric stretching vibrations of M–O bonds (M = Si and Al) in MO_4_ tetrahedral (Nasanjargal et al. 2021). The peaks at 789 and 468 cm^−1^ are ascribed to the stretching vibrations of M–O groups and the bending vibrations of O-M–O groups, respectively (Hommaid and Hamdo 2014). The spectrum of CTAB-zeolite, in contrast to natural zeolite, exhibits new absorption peaks located at 2919, 2853, and 1481 cm^−1^. The first and the second peaks correspond to the C-H asymmetric and symmetric stretching vibration modes of the methylene group of the surfactant (Nasanjargal et al. 2021). These two peaks together with that at 1481 cm^−1^, due to the C-N stretching vibrations of the quaternary ammonium group of the surfactant, testify that the natural zeolite’s surface is successfully covered by cetyltrimethylammonium bromide surfactant.

#### Dynamic light scattering

**Fig. 2:**
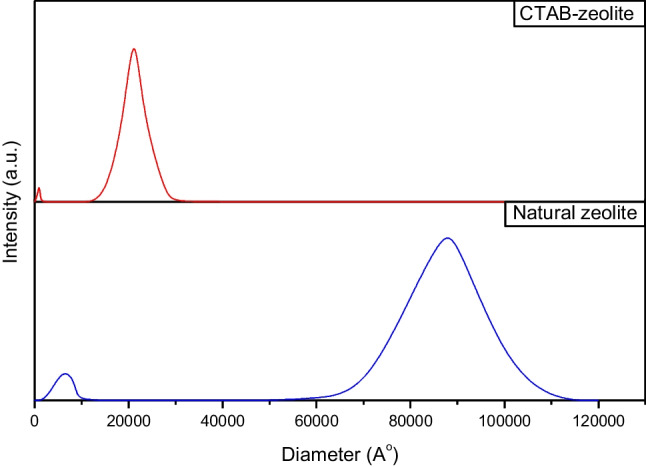
The intensity-based particle size distribution of natural zeolite and CTAB-zeolite

Owing to its fastness and simplicity, dynamic light scattering (DLS) is the most common and accurate technique for the analysis of particles (Guo et al. 2022). The DLS graphs of natural zeolite and CTAB-zeolite are shown in Fig. 2. This figure indicates that profiles of the studied materials exhibited two peaks of various nm-size ranges. The first peak of natural zeolite ranges between 2000 and 10,000 A° with a mean diameter of about 6000 A°, while the second one lies in the range 60,000–110,000 A° with a mean particle diameter of 87,100 A°. On the other hand, the first and second peaks of CTAB-zeolite are shifted to lower values that lie in the ranges 800–1400 A° and 15,000–35,000 A° with mean diameters of 940 and 21,200 A°, respectively. Based on these data, it can be concluded that surfactant-modified zeolite exhibited particle size smaller than natural zeolite. This finding is attributed to the occurrence of positively charged sites, trough adsorption of cetyltrimethylammonium (CTA^+^) cations onto the zeolite surface, which resulted in the stabilization of particles and restricted particle aggregation process.

#### Nitrogen adsorption–desorption isotherms

**Fig. 3:**
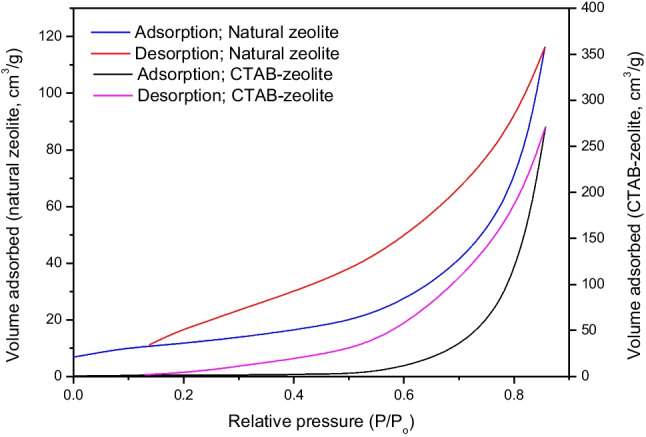
Nitrogen adsorption–desorption isotherms of zeolite before and after modification

According to the International Union of Pure and Applied Chemistry (IUPAC), the nitrogen adsorption–desorption isotherms and hysteresis loops in Fig. 3 revealed that natural zeolite and CTAB-zeolite shapes are corresponding to types IV and H3, respectively (Sing et al. 1985). This indicates the presence of slit-like mesopores in the studied materials. The specific surface areas of natural zeolite and CTAB-zeolite were calculated from the adsorption isotherms using the Brunauer–Emmett–Teller (EBT) equation, which is an important method for measuring the physisorption of gas molecules onto solid surfaces. The results clarified that the specific surface area of zeolite (*S*_EBT_ = 26.66 m^2^/g) is greatly reduced to 7.21 m^2^/g after modification with the surfactant. This reduction can be presumably attributed to the blockage of pores due to the adsorption of CTA^+^ molecules onto the zeolite surface (Li 1999). The pore volumes of natural zeolite and CTAB zeolite are found to be 0.029 and 0.033 cm^3^/g, respectively.

#### Pore size distribution

**Fig. 4:**
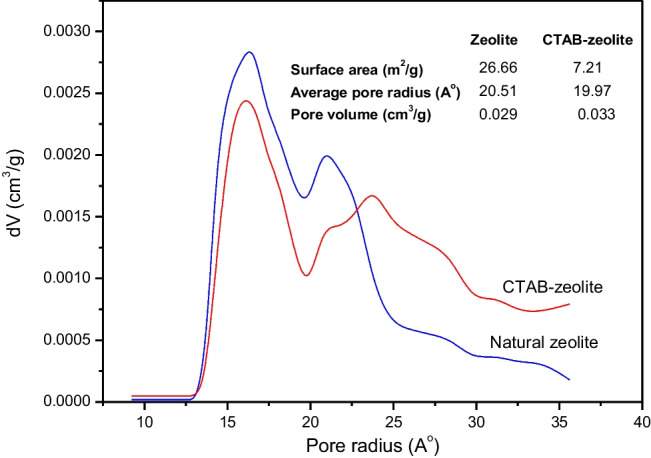
Pore size distribution of natural zeolite and CTAB-zeolite

The pore size distribution of natural zeolite before and after the modification process with cetyltrimethylammonium bromide was analyzed by the density functional theory (DFT) method, which is characterized by its accuracy for estimation of pore size (Soliman et al. 2019). The pore size distribution data depicted in Fig. 4 demonstrate that natural zeolite and CTAB-zeolite exhibited a non-uniform pore size in the range 15–25 A° and the calculated average pore radius was found to be 20.51 and 19.97 A°, respectively. Based on the predominant pore size, the porous materials are classified by the IUPAC into (Sing et al. 1985) microporous (< 20 A°), mesoporous (20–500 A°), and macroporous (> 500 A°). Accordingly, the data given in Fig. 4 suggested that natural zeolite and CTAB-zeolite had pores in the range of mesoporous materials. The insignificant change in the pore size of natural zeolite after modification indicated that surfactant molecules were adsorbed onto the surface of zeolite rather than inside its pores.

#### X-ray diffraction

**Fig. 5:**
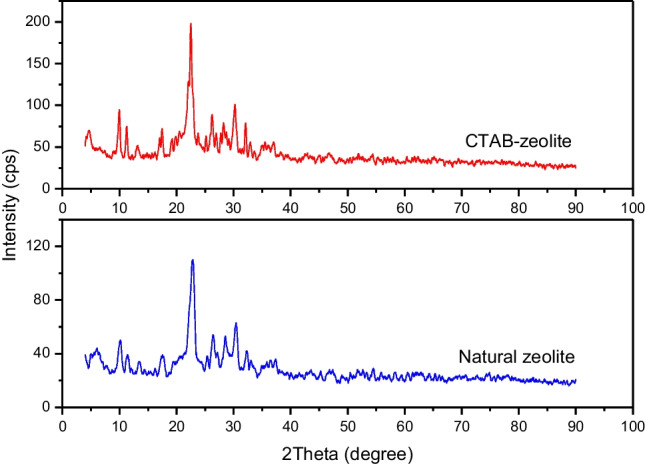
XRD diffractograms of natural zeolite and CTAB-zeolite

The XRD diffractograms of natural zeolite and modified zeolite are depicted in Fig. 5. This figure demonstrates that both of the studied zeolites show diffraction peaks at 4.7°, 9.8°, 11.3°, 13.3°, 17.6°, 22.6°, 26.2°, 28.5°, 30.3°, 32.1°, 33.1°, and 36.9°. This implies that there was no significant change in the diffraction peaks position of CTAB-zeolite. The data given in Fig. 5 further indicate that the peak intensities of CTAB-zeolite were lower than that of natural zeolite. Accordingly, it is deduced that (Nasanjargal et al. 2021**)**: (i) the zeolite’s surface is functionalized with cetyltrimethylammonium bromide surfactant, (ii) this modification process had no effect on the crystallinity of zeolite, and (iii) surfactant molecules are adsorbed onto zeolite surface rather than in its pores.

### Effect of modification

**Fig. 6:**
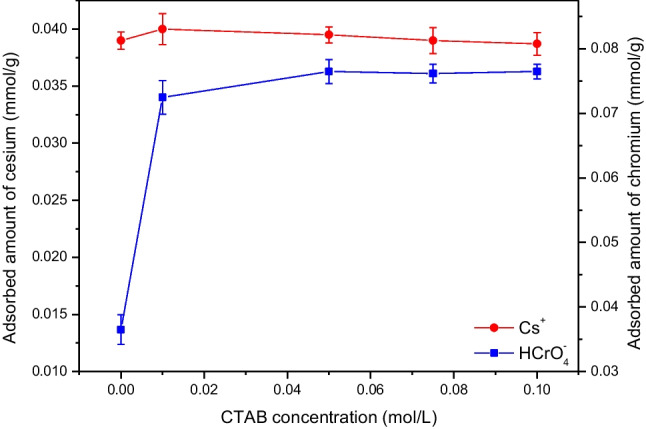
Adsorption efficiency of cesium and chromium onto zeolite modified at different CTAB concentration. [Cs^+^] = 0.25 mmol/L; [Cr(VI)] = 0.5 mmol/L; adsorbent mass = 6 g/L; pH = 3.2; contact time = 24 h

The influence of CTAB concentration (in the range 0–0.1 mol/L), loaded onto zeolite during its modification process, on the adsorption efficiency of 0.25 mmol/L Cs(I) and 0.5 mmol/L Cr(VI) using an adsorbent mass of 6 g/L is investigated at pH 3 and the results obtained are shown in Fig. 6. From this figure it can be seen that CTAB had no effect on the adsorption efficiency of Cs^+^ cations where an adsorbed amount of about 0.04 mmol/g is achieved at the studied surfactant concentrations. On the other hand, loading of CTAB onto zeolite is found to have an important role on the adsorption of the anionic species, HCrO_4_^−^. By increasing CTAB concentration, the amount of HCrO_4_^−^ adsorbed onto the modified zeolite is increased and reached its maximum value, 0.076 mmol/g, at concentrations ≥ 0.05 mol/L. Thereupon, zeolite was modified using a surfactant solution at a concentration of 0.075 mol/L and the resultant material, CTAB-zeolite, was utilized for conducting the subsequent adsorption experiments of Cs^+^ and HCrO_4_^−^ from aqueous solutions.

### Effect of the solution pH

**Fig. 7:**
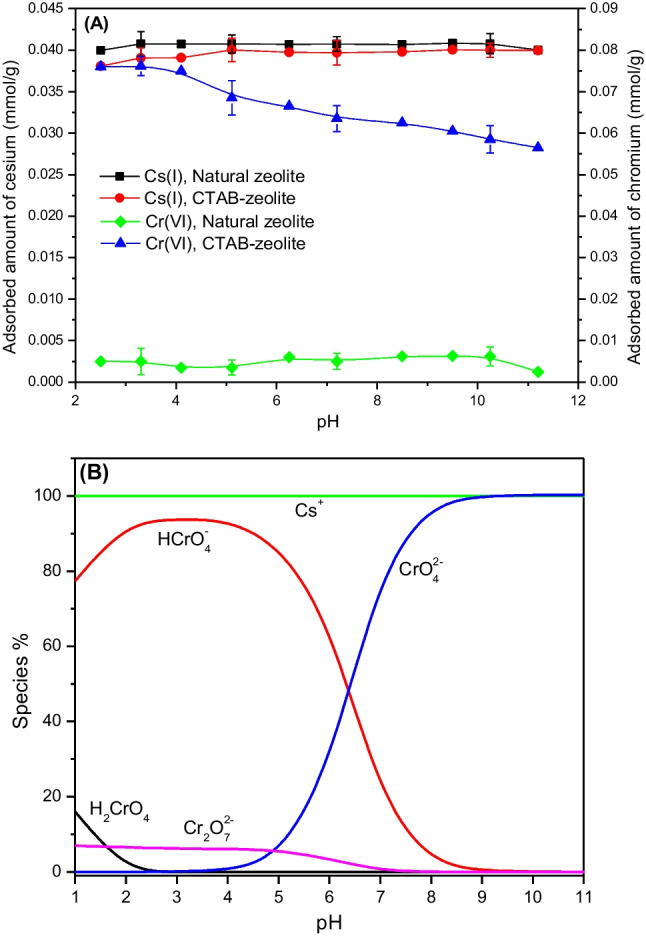
Effect of the solution pH on the adsorbed amounts of cesium and chromium using natural zeolite and CTAB-zeolite (**A**) and aqueous species of Cs(I) and Cr(VI) at different pH values (**B**). [Cs^+^] = 0.25 mmol/L; [Cr(VI)] = 0.5 mmol/L; adsorbent mass = 6 g/L; contact time = 24 h

The influence of the solution pH on the adsorbed amounts of 0.25 mmol/g cesium and 0.5 mmol/g chromium(VI), when coexisted in the aqueous solution, is studied in the pH range 2.5–11.2 using natural zeolite and CTAB-zeolite at an adsorbent mass of 6 g/L. The results obtained are presented in Fig. 7 A. As can be seen by this figure, neither the solution pH nor the modification process with cetyltrimethylammonium bromide surfactant had an effect on the adsorption efficiency of cesium. This is because the adsorbed amount of cesium onto natural zeolite and the modified one mostly remained unchanged with varying the solution pH in the studied range. It is well-recognized that zeolites possess a net negative structural charge, owing to the isomorphic substitution of tetravalent silicon (Si^4+^) atoms by trivalent aluminum (Al^3+^) atoms in their crystal lattice, which is compensated by exchangeable cations (e.g. Na^+^, K^+^, Ca^2+^, Mg^2+^) of alkali and alkaline earth metals (Zhang et al. 2021; Zeng et al. 2010). Based on this structure, zeolites exhibit mainly cation exchange properties and applied for adsorption of certain cations. Accordingly, the adsorbed amount of cesium achieved in this study either by natural or modified zeolite can be attributed to the replacement of exchangeable cations at the zeolite’s surface by Cs^+^ cations, which are the predominant species of cesium at the studied pHs.

For hexavalent chromium, the data given in Fig. 7 A reveal that utilization of natural zeolite resulted in low adsorption efficiency where adsorbed amounts below 0.007 mmol/g are obtained in the studied pH range, while an adsorbed amount of 0.076 mmol/g is achieved in the pH range 2.5–4.1 by using CTAB-zeolite which is decreased with further increase in the solution pH and reached 0.057 mmol/g at pH 11.2. To interpret these data, aqueous species of hexavalent chromium were calculated in the pH range 2–12 using the chemical software PHREEQC (Fig. 7 B). It is found that chromate monoacid is the predominant species (HCrO_4_^−^, ~ 96%) in the pH range 2.5–4.5 and the remainder percentage is due to the presence of Cr_2_O_7_^2−^ ion, whereas dichromate (CrO_4_^2−^) is the unique species in the pH range 8.5–12. In the pH range 4.5–8.5, both HCrO_4_^−^ and CrO_4_^2−^ ions coexisted in the aqueous solution (Szala et al. 2015):

$${\mathrm{H}}_{2}{\mathrm{CrO}}_{4} \leftrightarrow {\mathrm{HCrO}}_{4}^{-}+{\mathrm{H}}^{+}$$

$${\mathrm{HCrO}}_{4}^{-} \leftrightarrow {\mathrm{CrO}}_{4}^{2-}+{\mathrm{H}}^{+}$$

$${{\mathrm{Cr}}_{2}\mathrm{O}}_{7}^{2-}+{\mathrm{H}}_{2}\mathrm{O }\leftrightarrow {2\mathrm{HCrO}}_{4}^{-}$$

As shown, chromium(VI) ions present mainly as anionic species, HCrO_4_^−^ and/or CrO_4_^2−^, in the studied pH range of 2.5–11.2. The negatively charged surface of natural zeolite therefore had not the ability to effectively adsorb chromium(VI) anionic species due to charge repulsion. Modification of natural zeolite with cetyltrimethylammonium bromide (CTAB) surfactant alters the chemistry of the zeolite’s surface drastically due to the formation of positively charged admicelles at the zeolite’s surface, which are balanced by Br^−^ ions (Haggerty and Bowman 1994). Binding of HCrO_4_^−^ and CrO_4_^2−^ anions, which are the dominant species of chromium(VI) at the studied low and high pHs respectively, is thus governed by anion exchange of these species with Br^−^ counter-ions of CTA^+^ at the zeolite’s surface according to the following reactions (Warchol et al. 2006):

$$\mathrm{zeolite}-{\mathrm{CTA}}^{+}{\mathrm{Br}}^{-}+{\mathrm{HCrO}}_{4}^{-} \to \mathrm{ zeolite}-{\mathrm{CTA}}^{+}{\mathrm{HCrO}}_{4}^{-}+{\mathrm{Br}}^{-}$$

$$2(\mathrm{zeolite}-{\mathrm{CTA}}^{+}{\mathrm{Br}}^{-})+{\mathrm{CrO}}_{4}^{2-} \to (\mathrm{zeolite}-{\mathrm{CTA}}^{+}{)}_{2}-{\mathrm{CrO}}_{4}^{2-}+{2\mathrm{Br}}^{-}$$

Regarding these equations, it can be observed that the adsorption of CrO_4_^2−^ at high pH values (particularly at pHs > 7) is governed by the consumption of a higher number of adsorption sites. This reason together with the competition between OH^−^ and CrO_4_^2−^ ions for binding with the positively charged quaternary ammonium groups of the surfactant at the zeolite’s surface caused the reduction in the adsorbed amount of chromium(VI) in the alkaline environment (Fig. 7 A).

### Effect of CTAB-zeolite mass

**Fig. 8:**
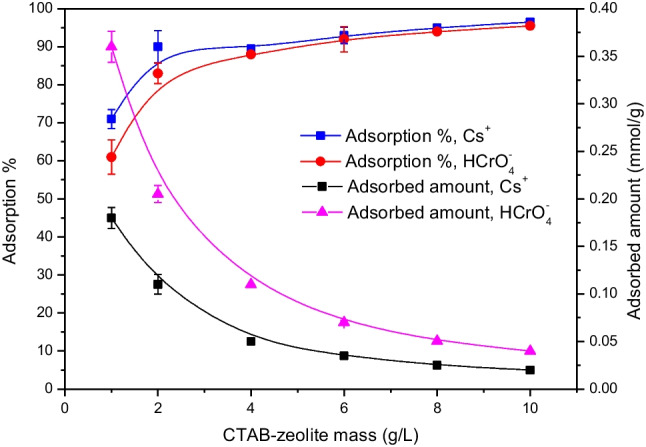
Effect of CTAB-zeolite mass on the adsorption % and the adsorbed amounts of cesium and chromium. [Cs^+^] = 0.25 mmol/L; [Cr(VI)] = 0.5 mmol/L; pH = 3.2; contact time = 24 h

It is generally important, from the economic and waste management points of view, to determine the lowest adsorbent mass needed to obtain the highest removal efficiency of (radio)toxicants. Consequently, the impact of CTAB-zeolite mass in the range 1–10 g/L on the removal percentages and the adsorbed amounts of Cs^+^ and HCrO_4_^−^ ions is studied at pH 3 and the data obtained are shown in Fig. 8. This figure shows that both the removal percentage and the adsorbed amount of the concerned (radio)toxicants are greatly dependent on the adsorbent mass. The adsorption percentage is increased with increasing the mass of CTAB-zeolite and reached values of more than 92% at adsorbent masses ≥ 6 g/L. This enhancement in the adsorption percentage is ascribed to the plenty of functional groups, exchangeable cations, and ammonium groups of CTA^+^, at CTAB-zeolite at which Cs^+^ cations and HCrO_4_^−^ anions are adsorbed, respectively. On the other hand, the adsorbent mass is found to have a deleterious effect on the adsorbed amounts of the studied (radio)toxicants. By increasing the mass of CTAB-zeolite from 1 to 10 g/L, the adsorbed amount of Cs^+^ is reduced from 0.18 to 0.02 mmol/g while that of HCrO_4_^−^ is reduced from 0.36 to 0.04 mmol/g. This reduction in the adsorbed amounts is attributed to (i) the decrease of CTAB-zeolite surface area and the increase of the diffusion path length owing to particle aggregation (Hamoud et al. 2021; Mahmoud et al. 2019; Chen et al. 2012), and (ii) the utilization of unsaturated adsorption sites, of the extra CTAB-zeolite particles, which is accounted during estimating the adsorbed amounts of the studied (radio)toxicants (Mahmoud et al. 2019; Xing et al. 2011). During the calculation of the adsorbed amounts of Cs^+^ and HCrO_4_^−^ ions using Eq. (3), it is found that the slight increase in the adsorption percentage particularly at CTAB-zeolite masses ≥ 2 g/L did not compensate for the decrease in the adsorbed amounts caused by the significant increase in the adsorbent mass. Taking the economic and waste management points of view into consideration, an adsorbent mass of 6 g/L was found to be the most appropriate one for further adsorption experiments conducted for Cs^+^ and HCrO_4_^−^ ions in this investigation.

### Effect of contact time and modeling of data

**Fig. 9:**
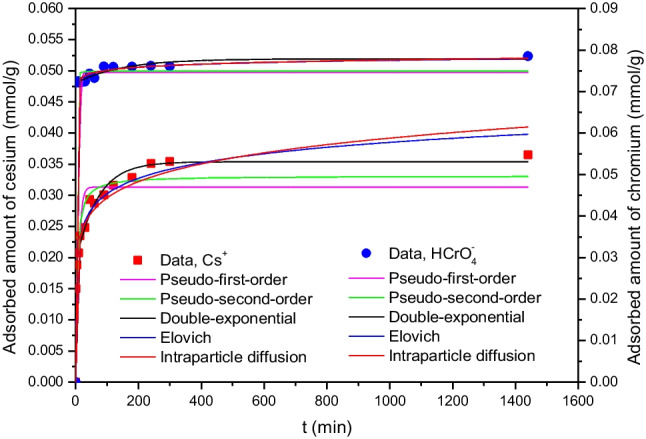
Effect of contact time on the adsorbed amounts of cesium and chromium and modeling of the data using various kinetic models using the non-linear method of analysis. *[*Cs^+^] = 0.25 mmol/L; [Cr(VI)] = 0.5 mmol/L; adsorbent mass = 6 g/L; pH = 3.2

The effect of contact time, in the range 0–1440 min, on simultaneous adsorption of Cs^+^ and HCrO_4_^−^ ions onto 6 g/L CTAB-zeolite is studied at pH 3.2 and the results obtained are represented in Fig. 9. The data depicted in this figure indicate that the adsorbed amounts are sharply increased with time where 70% of Cs^+^ and 90% of HCrO_4_^−^ are removed in the first hour. Further increase in contact time increased the adsorbed amount of Cs^+^ gradually, while slightly improving that of HCrO_4_^−^ and the equilibrium is attained at 80 and 240 min, respectively. However, the adsorption experiments of this study were conducted at 24 h to ensure equilibration.

For estimating the kinetic parameters, which are useful for designing an effective adsorption process, the kinetic data of Cs^+^ and HCrO_4_^−^ ions are analyzed by five adsorption kinetic models using the non-linear analysis method based on OriginPro 8.5 software. These models are pseudo-first-order (Eq. 8), pseudo-second-order (Eq. 9), double-exponential (Eq. 10), Elovich (Eq. 11), and intraparticle diffusion (Eq. 12). Non-linear equations of these models are expressed as (Mahmoud et al. 2014, 2019; Ho and McKay 1999; Weber and Morris 1963; Lagergren 1898):

8 $${q}_{t}={q}_{\mathrm{e}}\left(1-{e}^{{-K}_{1}t}\right)$$

9 $${q}_{t}={q}_{\mathrm{e}}\left(\frac{{K}_{2}{q}_{\mathrm{e}}t}{1+{K}_{2}{q}_{\mathrm{e}}t}\right)$$

10 $${q}_{t}={q}_{0}+{q}_{\mathrm{e}1}\left(1-{e}^{{-K}_{3}t}\right)+{q}_{\mathrm{e}2}\left(1-{e}^{{-K}_{4}t}\right)$$

11 $${q}_{t}=\left(\frac{1}{\beta }\right)Ln \left(1+\alpha \beta t\right)$$

12 $${q}_{t}={K}_{5}{t}^{m}$$

where *q*_e_ and *q*_*t*_ are the amount of adsorbate (mmol/g) adsorbed at equilibrium and at time *t*, respectively. The parameters *K*_1_ (min^−1^), *K*_2_ (g/mmol min), *K*_3_ (min^−1^), *K*_4_ (min^−1^), and *K*_5_ (mmol/g min^0.5^) are the rate constants. For Elovich kinetic model, *α* is the initial adsorption rate (mmol/g min) and *β* is the desorption constant (g/mmol) and it is related to the surface coverage and activation energy of chemical adsorption (Mahmoud et al. 2014). For intraparticle diffusion to be the rate-controlling step, the value of the non-dimensional coefficient, *m*, should be equal 0.5 (Mahmoud et al. 2019). Non-linear fittings of the kinetic data of the studied (radio)toxicants to the abovementioned kinetic models are shown in Fig. 9. The calculated values of kinetic parameters, correlation coefficient (*R*^2^), residual sum of square (RSS), and chi-square (*χ*^2^) are tabulated in Table 1. This table demonstrates that the studied kinetic models had the ability to well-fit the kinetic data of HCrO_4_^−^ where high correlation coefficient values (*R*^2^ ≈ 0.99) are obtained. Among the studied kinetic models, only the double-exponential and Elovich ones succeeded to fit the kinetic data of Cs^+^ (*R*^2^ > 0.98). To determine the most appropriate kinetic model for describing the present kinetic data, the RSS and *χ*^2^ values are taken into consideration. By comparing values of these error functions, it can be seen that the double-exponential model exhibited the lowest values. This finding indicates that the double-exponential model can be considered the best kinetic model for describing the kinetic data of Cs^+^ and HCrO_4_^−^. The consistency of the *q*_e_ value computed from the double-exponential model (*q*_e_ = 0.036 and 0.078 mmol/g for Cs^+^ and HCrO_4_^−^, respectively), which is the sum of *q*_e1_ and *q*_e2_, with the experimental values (Table 1) is a further confirmation of the vantage of the double-exponential model for representing the kinetic data of Cs^+^ and HCrO_4_^−^. Regarding the K_3_ values of this model, it can be observed that HCrO_4_^−^ exhibited greater value (*K*_3_ = 3.37 × 10^3^ min^−1^) than Cs^+^ (*K*_3_ = 0.015 min^−1^). These values pointed out that the anionic species are adsorbed onto the surface of CTAB-zeolite, while adsorption of the cationic ones took place at the interior sites of the adsorbent.

**Table 1:** Kinetic parameters for adsorption of Cs^+^ and HCrO_4_^−^ onto CTAB-zeolite

Model	Parameter	Value	
		Cs^+^	HCrO_4_^−^
Pseudo-first-order	*K*_1_ (min^−1^)	0.139	2.261
	*q*_e_ (mmol/g)	0.031	0.075
	*R*^2^	0.8047	0.9899
	*χ*^2^	1.96 × 10^−5^	4.05 × 10^−6^
	*RSS*	2.35 × 10^−4^	4.86 × 10^−5^
Pseudo-second-order	K_2_ (g/mmol min)	6.596	157.09
	*q*_e_ (mmol/g)	0.033	0.074
	*R*^2^	0.9062	0.9916
	*χ*^2^	9.41 × 10^−6^	3.35 × 10^−6^
	*RSS*	1.13 × 10^−4^	4.02 × 10^−5^
Double-exponential	*q*_0_ (mmol/g)	3.83 × 10^−5^	5.24 × 10^−6^
	*q*_1_ (mmol/g)	0.019	0.006
	*q*_2_ (mmol/g)	0.017	0.072
	*K*_3_ (min^−1^)	0.015	3.37 × 10^3^
	*K*_4_ (min^−1^)	0.942	0.007
	*R*^2^	0.9813	0.9981
	*χ*^2^	1.47 × 10^−6^	6.52 × 10^−7^
	*RSS*	1.39 × 10^−5^	5.86 × 10^−6^
Elovich	*α* (mmol/g min)	0.139	1.95 × 10^4^
	*β* (g/mmol)	271.52	958.6
	*R*^2^	0.9852	0.9981
	*χ*^2^	1.88 × 10^−6^	9.57 × 10^−7^
	*RSS*	1.77 × 10^−5^	7.12 × 10^−6^
Intraparticle diffusion	*K*_5_ (mmol/g min^0.5^)	0.017	0.071
	*m*	0.126	0.014
	*R*^2^	0.9637	0.9982
	*χ*^2^	3.46 × 10^−6^	7.28 × 10^−7^
	RSS	4.37 × 10^−5^	8.74 × 10^−6^
Experimental *q*_e_ (mmol/g)		0.036	0.078

Generally, the adsorption process of an adsorbate onto an adsorbent by the batch equilibration method proceeds through three steps (Mahmoud et al. 2014): (i) mass transfer of the adsorbent from the bulk solution to the external surface of the adsorbent, (ii) diffusion of the adsorbate to the internal adsorption sites, and (iii) equilibrium adsorption. To distinguish between the external mass transfer and the internal diffusion, the kinetic data are further analyzed by the intraparticle diffusion model (Eq. 12). Fitting of the kinetic data of Cs^+^ and HCrO_4_^−^ to this diffusion model resulted in *R*^2^ values of 0.9637 and 0.9882, respectively. These satisfactory values show the ability of this model to describe the present kinetic data with *m* values below 0.5, indicating that the intraparticle diffusion was not the sole rate-controlling step in the adsorption process of Cs^+^ and HCrO_4_^−^ onto CTAB-zeolite.

### Adsorption isotherms and modeling of data

**Fig.10:**
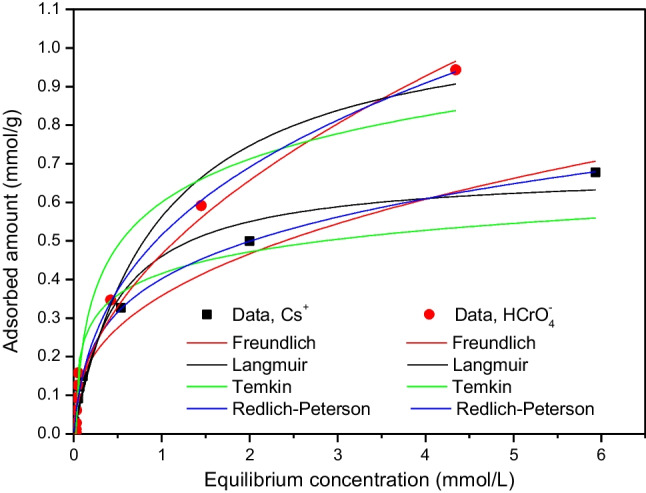
Adsorption isotherms of cesium and chromium and their modeling using different isotherm models by the non-linear regression analysis method. Adsorbent mass = 6 g/L; pH = 3.2; contact time = 24 h

Figure 10 illustrates the relation between the equilibrium concentrations of the concerned (radio)toxicants and their adsorbed amounts per gram of CTAB-zeolite at constant pH and temperature values of 3.2 and 25 °C, respectively. As shown by this figure, the adsorbed amounts of Cs^+^ and HCrO_4_^−^ are increased with increasing their equilibrium concentration, which is ascribed to the increase in their mass driving force toward the adsorption sites at CTAB-zeolite.

In reviewing the literature, it is found that several theoretical and empirical relations have been used for modeling the adsorption isotherms. Four well-recognized adsorption isotherm models are used in the current study to analyze the adsorption isotherms of Cs^+^ and HCrO_4_^−^ onto CTAB-zeolite. These models are Freundlich (Eq. 13), Langmuir (Eq. 14), Temkin (Eq. 15), and Redlich-Peterson (Eq. 16), which are represented by the following equations (Mahmoud et al. 2019; Temkin and Pyzhev 1940; Langmuir 1918; Freundlich 1906):

13 $${q}_{\mathrm{e}}={K}_{F}{C}_{\mathrm{e}}^{1/n}$$

14 $${q}_{e}=\frac{{q}_{m}{K}_{L}{C}_{\mathrm{e}}}{1+{K}_{L}{C}_{\mathrm{e}}}$$

15 $${q}_{e}={B}_{T}Ln {{A}_{T}C}_{\mathrm{e}}$$

16 $${q}_{e}=\frac{{K}_{R}{C}_{\mathrm{e}}}{1+{b}_{R}{C}_{\mathrm{e}}^{g}}$$

where q_e_ is the adsorbed amount of (radio)toxicants at equilibrium (mmol/g), q_m_ is the maximum adsorption capacity (mmol/g) of CTAB-zeolite for Cs^+^ and HCrO_4_^−^ and C_e_ is their equilibrium concentration (mmol/L). Non-linear fittings of the adsorption isotherm data of Cs^+^ and HCrO_4_^−^ onto CTAB-zeolite to the studied adsorption isotherm models (Eqs. 13–16) are shown in Fig. 10. The obtained values of *R*^2^, isotherm parameters (*K*_F_, *n*, *K*_L_, *q*_m_, *A*_T_, *B*_T_, *K*_R_, *b*_R_, and *g*), RSS and *χ*^2^ are given in Table 2. It can be observed that the Redlich-Peterson model did not only exhibit the highest correlation coefficient values (*R*^2^ = 0.9993 for Cs^+^ and 0.9807 for HCrO_4_^−^), but also the lowest RSS and *χ*^2^ values. Consequently, adsorption isotherms of Cs^+^ and HCrO_4_^−^ are better fitted by the Redlich-Peterson equation. This hybrid isotherm model describes the adsorption equilibria over a wide range of concentrations and applies to homogeneous and heterogeneous systems. The g value of the Redlich-Peterson model in the present study (Table 2) lies between 0 and 1 (*g* = 0.751 and 0.664 for Cs^+^ and HCrO_4_^−^, respectively), suggesting favorable adsorption of (radio)toxicants onto CTAB-zeolite (Mahmoud et al. 2019).

**Table 2:** Isotherm parameters for adsorption of Cs^+^ and HCrO_4_^−^ onto CTAB-zeolite

Model	Parameter	Value	
		Cs^+^	HCrO_4_^−^
Freundlich	*K*_F_ (mmol^1−n^L^n^/g)	0.358	0.465
	*n*	2.620	2.013
	*R*^2^	0.9845	0.9733
	*χ*^2^	8.12 × 10^−3^	2.53 × 10^−3^
	RSS	6.50 × 10^−4^	2.03 × 10^−2^
Langmuir	*K*_L_ (L/mmol)	2.058	1.028
	q_m_ (mmol/g)	0.684	1.180
	*R*^2^	0.9746	0.9638
	*χ*^2^	1.34 × 10^−3^	3.43 × 10^−3^
	RSS	1.07 × 10^−2^	2.75 × 10^−2^
Temkin	*A*_T_ (L/g)	174.12	40.633
	*B*_T_ (kJ/mol)	0.081	0.162
	*R*^2^	0.8753	0.9578
	*χ*^2^	6.55 × 10^−3^	4 × 10^−3^
	RSS	5.24 × 10^−2^	3.19 × 10^−2^
Redlich-Peterson	*K*_R_ (L/g)	3.809	3.19
	*b*_R_ (L/mmol)	8.476	5.199
	g	0.751	0.664
	*R*^2^	0.9993	0.9807
	*χ*^2^	3.63 × 10^−5^	1.8 × 10^−3^
	RSS	2.54 × 10^−4^	1.28 × 10^−2^

### Effect of temperature and estimating thermodynamic parameters

**Fig. 11:**
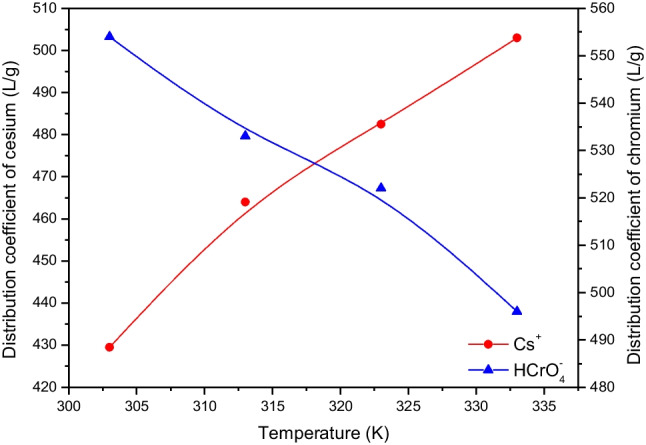
Effect of temperature on the distribution coefficient values of cesium and chromium. [Cs^+^] = [Cr(VI)] = 2.5 mmol/L; adsorbent mass = 6 g/L; pH = 3.2;contact time = 24 h

Figure 11 shows the effect of temperature on the adsorption efficiency of Cs^+^ cations and HCrO_4_^−^ anions onto CTAB-zeolite. As can be seen by the data given in this figure, the distribution coefficient of cesium is directly proportional to temperature where it is increased from 430 to 503 mL/g by elevating temperature from 303 to 333 K. On the contrary, temperature is found to have a negative effect on the distribution coefficient value of chromium as it is decreased from 555 to 496 mL/g with increasing temperature from 303 to 333 K. Based on the data depicted in Fig. 11, the value of free energy change (∆G°) can be estimated using the following relation (Hamoud et al. 2021):

17 $$\Delta {G}^{^\circ }=-RTln{K}_{d}$$

where *R* is the universal gas constant (*R* = 0.008314 kJ/mol) and *T* is the absolute temperature in Kelvin. The obtained data tabulated in Table 3 indicate that Cs^+^ and HCrO_4_^−^ ions exhibited ∆*G*° in the range − 17.094 to − 15.174 kJ/mol and − 15.942 to − 17.232 kJ/mol, respectively, in the studied temperature range of 303–333 K. The negative sign of ∆*G*° values suggested that the adsorption of Cs^+^ and HCrO_4_^−^ ions onto CTAB-zeolite was spontaneous processes. Where ∆*G*° of Cs^+^ is shifted to less negative values and that of HCrO_4_^−^ are shifted to more negative values with increasing temperature from 303 to 333 K, the adsorption process of the cationic species is more spontaneous at low temperatures while that of the anionic species is more spontaneous at high temperatures. According to the literature, the magnitude of ∆*G*° can provide information to differentiate between physical and chemical adsorption processes (Petrucci and Harwood 1997). The species is adsorbed onto the solid surface by the physical process if ∆*G*° ranges between 0 and − 20 kJ/mol. While for chemical adsorption, ∆*G*° ranges between − 80 and − 400 kJ/mol. Accordingly, the studied species are physically adsorbed onto CTAB-zeolite and the system does not gain energy from the surroundings.

**Table 3:** Thermodynamic parameters for adsorption of Cs^+^ and HCrO_4_^−^ onto CTAB-zeolite

Adsorbate	Temperature (K)	∆*G*° (kJ/mol)	∆*H*° (kJ/mol)	∆*S*° (kJ/mol K)	*R*^2^
Cs^+^	303	− 17.094	4.218	0.064	0.9571
	313	− 16.454			
	323	− 15.814			
	333	− 15.174			
HCrO_4_^−^	303	− 15.942	− 2.913	0.043	0.9594
	313	− 16.372			
	323	− 16.802			
	333	− 17.232			

The other thermodynamic parameters, enthalpy change (∆*H*°) and entropy change (∆*S*°), are calculated using the following equation (Hamoud et al. 2021):

18 $$n{K}_{\mathrm{d}}=\frac{{\Delta S}^{o}}{R}-\frac{\Delta {H}^{o}}{RT}$$

The plot of *lnK*_d_ versus 1/T for Cs^+^ and HCrO_4_^−^ ions, figures not shown, yields straight lines with high correlation coefficients (*R*^2^ > 0.95) from which ∆*H*° and ∆*S*° are calculated and recorded in Table 3. The positive value of entropy change (∆*H*° = 4.218 kJ/mol) suggested that adsorption process of Cs^+^ cations onto the modified zeolite was endothermic in nature and heat is gained from the surroundings. For HCrO_4_^−^ anions, ∆H° had a negative value (− 2.913 kJ/mol) indicating that the adsorption process was exothermic. These low values of ∆*H*° further confirmed that both Cs^+^ and HCrO_4_^−^ ions are physically adsorbed onto CTAB-zeolite. Finally, the positive values of entropy change (∆*S*° = 0.064 and 0.043 kJ/mol K for Cs^+^ and HCrO_4_^−^, respectively) indicate the increased randomness at the solid–liquid interface during the adsorption processes of the studied species.

### Effect of coexisting ions

**Fig. 12:**
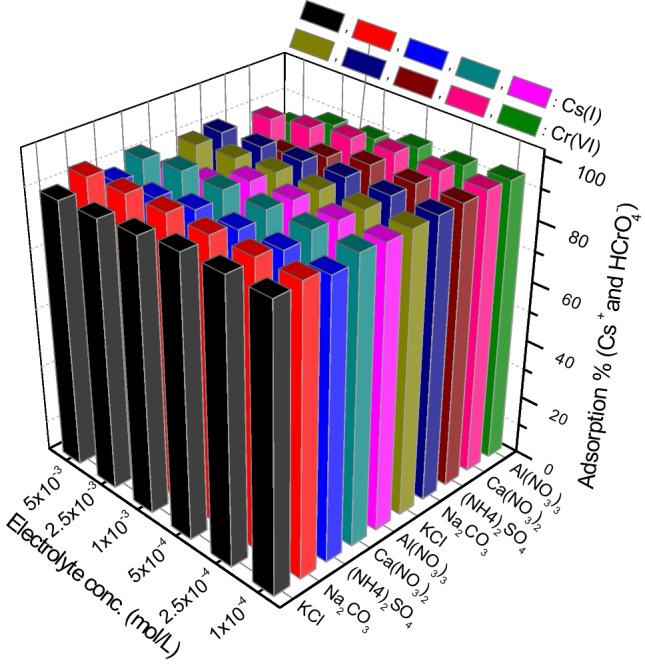
Effect of foreign ions type and concentration on the adsorption percentages of cesium and chromium. [Cs^+^] = 0.25 mmol/L; [Cr(VI)] = 0.5 mmol/L; adsorbent mass = 6 g/L; pH = 3.2; contact time = 24 h

Generally, the effect of coexisting ions is one of the most important variables during studying the adsorption process of metal ions from aqueous solutions. Based on this parameter, the ability of the adsorbent to remove (radio)toxicants from actual liquid wastes containing foreign cations and/or anions could be evaluated. Therefore, the impact of the coexistence of various cations (K^+^, Na^+^, NH_4_^+^, Ca^2+^, and Al^3+^) and anions (Cl^−^, NO_3_^−^, CO_3_^2−^, and SO_4_^2−^) on the adsorption percentages of Cs^+^ cations and HCrO_4_^−^ anions from their binary systems was investigated by conducting the adsorption experiments from different electrolyte solutions in the concentration range 1 × 10^−4^ to 5 × 10^−3^ mol/L using CTAB-zeolite mass of 6 g/L. The results obtained (Fig. 12) illustrate that the existence of KCl, Na_2_CO_3_, and Ca(NO_3_)_2_ had no effect on the adsorption percentages of Cs^+^ and HCrO_4_^−^ ions at the studied concentrations. Whereas, a slight deleterious effect (~ 15%) on the adsorption efficiency of the studied (radio)toxicants is observed in presence of the other electrolytes, (NH_4_)_2_SO_4_ and Al(NO_3_)_3_, particularly at concentrations higher than 1 × 10^−3^ mol/L. The data given in Fig. 12 obviously confirmed that the surfactant-modified zeolite, synthesized in this study, had the ability to effectively adsorb both Cs^+^ cations and HCrO_4_^−^ anions by a one-step process from solutions having high concentrations of foreign ions. Thus, it can be considered a promising material for simultaneous adsorption of cationic and anionic (radio)toxicants that coexisted in the aqueous solution with high concentrations of background electrolytes.

### Maximum adsorption capacity and comparison with other studies

**Fig. 13:**
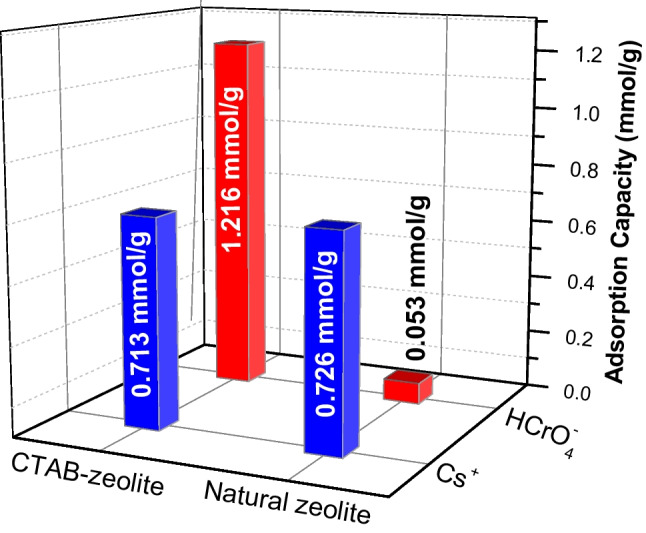
Maximum adsorption capacities of natural zeolite and CTAB-zeolite for cesium and chromium. Adsorbent mass = 10 g/L; pH = 3; contact time = 24 h

The maximum adsorption capacity of natural zeolite and CTAB-zeolite, experimentally determined at pH 3, toward Cs^+^ and HCrO_4_^−^ ions are shown in Fig. 13. This figure indicates that the modification process of zeolite with CTAB had no effect on the adsorption efficiency of Cs^+^ where maximum adsorption capacity (*Q*_max_) of 0.726 and 0.713 mmol/g are achieved using natural zeolite and CTAB-zeolite, respectively, while for HCrO_4_^−^, the maximum adsorption capacity of CTAB-zeolite (*Q*_max_ = 1.216 mmol/g) is found to be 23 times greater than that of natural zeolite (*Q*_max_ = 0.053 mmol/g). To further confirm the efficiency of CTAB-zeolite for the simultaneous removal of Cs^+^ and HCrO_4_^−^ ions, its maximum adsorption capacities are compared with those reported in the literature (Table 4). The data given for Cs^+^ in this table show that most of the compared materials had adsorption capacity in the range 0.115–0.583 mmol/g, which are lower than that achieved by CTAB-zeolite. Except for nano-malachite, the adsorption capacity of the employed adsorbent in this study for Cr(VI) is much greater than those obtained by the other adsorbents. Although nano-malachite had greater adsorption capacity (*Q*_max_ = 1.612 mmol/g) than CTAB-zeolite, it is limited in practical application owing to its small size and difficulty in subsequent separation of the solid phase. According to the values recorded in Table 4, it can be concluded that CTAB-zeolite had the ability to simultaneously adsorb Cs^+^ and HCrO_4_^−^ ions with high capacity and thus its potentiality for removal of cationic and anionic species by one-step process from aqueous solutions.

**Table 4:** Adsorption capacities for Cs(I) and Cr(VI) using different adsorbents in comparison with those obtained in the present study using CTAB-zeolite

Adsorbate	Comments	Adsorbent	*Q*_max_ (mmol/g)	Reference
Cs(I)	pH = 5, contact time = 1 h	Activated Al_2_O_3_	0.825	Hassan et al. 2019
	Contact time = 2 h, nitric acid media	P(AA-MA)/Al_2_O_3_-SiO_2_	0.121	Attallah et al. 2016
	Contact time = 24 h, Cs^+^ is coexisted with Sr^2+^	PB-HAP-MAs	0.186	Park et al. 2021
	pH = 6, contact time = 2 h	Nano-Fe/Cu-zeolite	0.583	Eljamal et al. 2019
	Contact time = 2 h	Nanocrystalline mordenite	0.281	Lee et al. 2016
	pH = 4, contact time = 2 h	Modified SSM slag	0.394	Khandaker et al. 2018
	pH = 3.4, contact time = 2 h, Cs(I) is coexisted with Cr(VI)	PAN/ferrocyanide composite	0.317	Hamoud et al. 2020
	pH = 6, contact time = 24 h	Mesoporous geopolymers	0.115	Lee et al. 2017
	pH = 9.4, contact time = 2 h	Magnetite	0.532	Sheha and Metwally 2007
	contact time = 2 h	Bio-slag	0.384	Khandaker et al. 2020
	pH = 3, contact time = 24 h, Cs^+^ is coexisted with Cr(VI)	CTAB-zeolite	0.713	Current work
Cr(VI)	pH = 1.5, contact time = 7 h	HDTMA-zeolite	0.028	Hommaid and Hamdo 2014
	pH = 5, contact time = 24 h	HDPB-zeolite	0.281	Zeng et al. 2010
	pH = 6.9, contact time = 24 h	CPB-zeolite	0.018	Ghiaci et al. 2004
	pH = 4, contact time = 24 h	ODTMA-zeolite	0.237	Szala et al. 2015
	pH = 7, contact time = 24 h	HDTMA-zeolite	0.41	Swarnkar and Radha 2011
	pH = 3.4, contact time = 2 h, Cr(VI) is coexisted with Cs^+^	PAN/ferrocyanide composite	0.381	Hamoud et al. 2020
	pH = 3.9, contact time = 5 h, Cr(VI) is coexisted with Co^2+^	HQS/MCM-41	0.596	Soliman et al. 2019
	pH = 5, contact time = 24 h, Cr(VI) is coexisted with Cu^2+^	HDTMA-zeolite	0.0093	Zekavat et al. 2020
	pH = 4, contact time = 48 h	Pb-modified zeolite	0.371	Thanos et al. 2017
	pH = 5.5, contact time = 1 h	HDTMA-kaolinite	0.545	Jin et al. 2014
	pH = 4.5	CPB-montmorillonite	0.354	Brum et al. 2010
	pH = 4, contact time = 12 h	Nano-malachite	1.612	Saikia et al. 2010
	pH = 7, contact time = 24 h	Zeolite/ZnAl-LDH	0.005	Zhang et al. 2021
	pH = 3, contact time = 0.5 h	Activated carbon	0.849	Wang et al. 2018
	pH = 1, contact time = 18 h	Saw dust	0.814	Gupta and Babu 2009
	pH = 3, contact time = 24 h, is Cr(VI) coexisted with Cs^+^	CTAB-zeolite	1.216	Current work

*PB-HAP-Mas*, Prussian blue-hydroxyapatite-embedded micro-adsorbents; *P(AA-MA)*, poly(acrylic acid-maleic acid), *HDTMA*, hexadecyltrimethylammonium bromide; *HDPB*, hexadeylpyridinium bromide; *CPB*, cetylpyridinium bromide; *PAN*, polyacrylonitrile; *ODTMA*, octadecyltrimethylammonium bromide; *HQS*, 8-hydroxyquinoline-5-sulfonic acid

### Desorption study

**Fig. 14:**
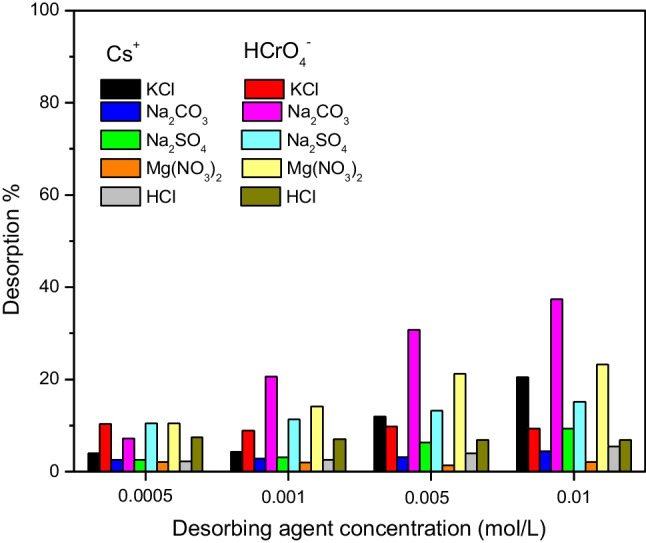
Desorption of cesium and chromium loaded onto CTAB-zeolite using different desorbing agents at various concentrations

CTAB-zeolite is efficiently utilized in the present study as an adsorbent for the simultaneous removal of ^134^Cs^+^ and HCrO_4_^−^ from aqueous solutions. At radioactive waste management facilities, the solid phase extractor should have the ability to strongly attract the radionuclides to prevent their release and diffusion in the environment. Therefore, it was important to further the binding strength of the concerned (radio)toxicants being adsorbed at the employed adsorbent. To achieve this goal, desorption of Cs^+^ and HCrO_4_^−^ loaded onto CTAB-zeolite is studied using numerous desorbing agents (KCl, Na_2_CO_3_, Na_2_SO_4_, Mg(NO_3_)_2_ and HCl) at different concentrations in the range 5 × 10^−4^ to 1 × 10^−2^ mol/L. The obtained results are illustrated in Fig. 14. This figure indicates that maximum desorption percentages of about 23 (for ^134^Cs^+^) and 36% (for HCrO_4_^−^) are obtained at the higher studied concentrations of KCl and Na_2_CO_3_, respectively, whereas the other tested desorbing agents mostly failed to desorb the concerned (radio)toxicants where desorption percentages ≤ 10% are obtained. Desorption data given in Fig. 14 show that Cs^+^ and HCrO_4_^−^ are strongly adsorbed onto CTAB-zeolite and thus suggest its potential application in waste management of radioactive wastes for simultaneous adsorption of anionic and cationic radionuclides.

### Proposed adsorption mechanism

**Fig. 15:**
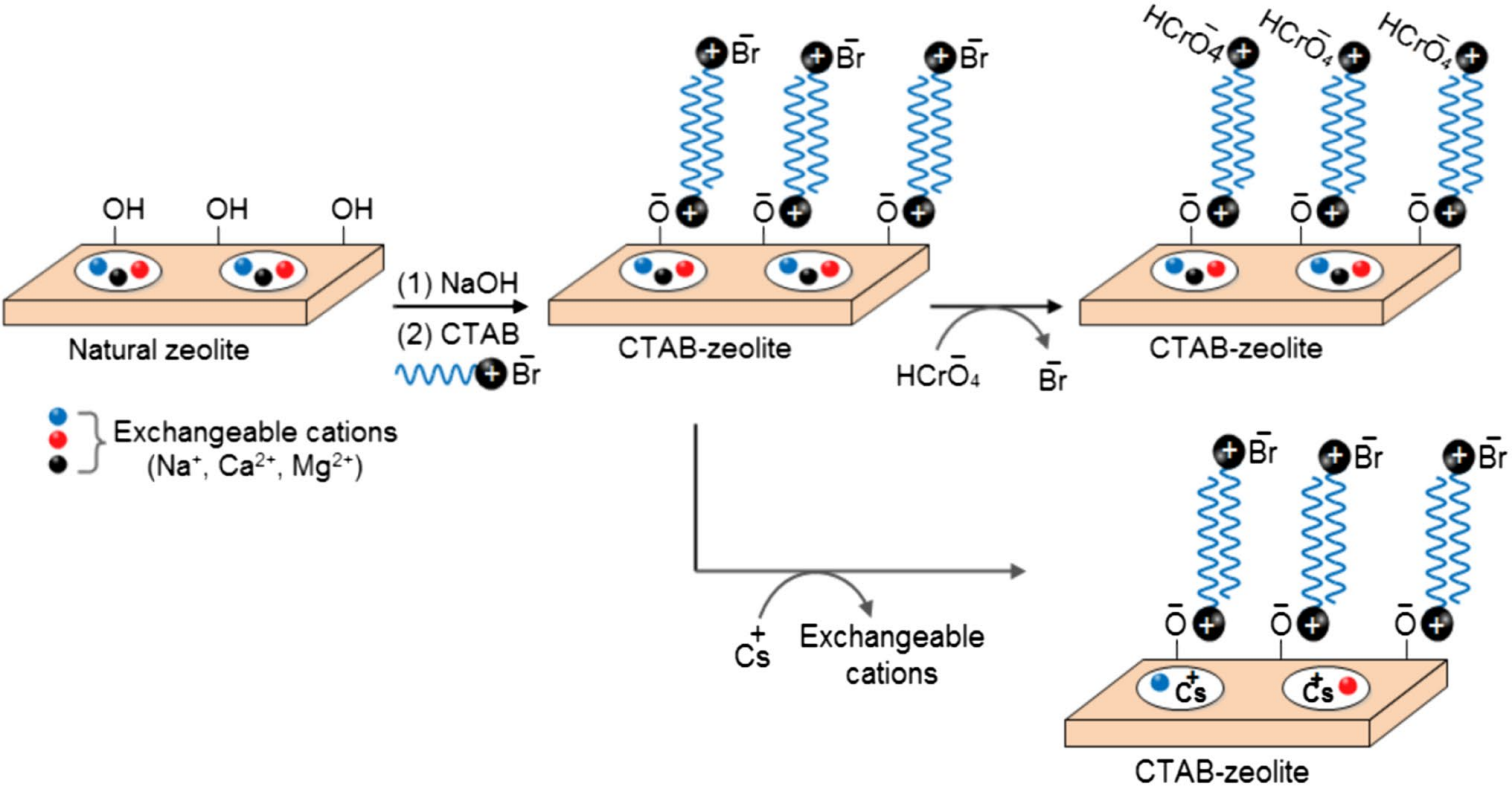
The proposed adsorption mechanism of Cs^+^ and HCrO_4_^−^ onto CTAB-zeolite

Taking into consideration the aqueous speciation of cesium and hexavalent chromium at the optimum pH range of 2.5–4.2, the characterization of zeolite before and after the organo-modification process and the results achieved in this investigation which are discussed in the following lines, the adsorption mechanism of the concerned (radio)toxicants is proposed (Fig. 15). The data obtained by the PHREEQC program (Fig. 7 B) revealed that Cs^+^ and HCrO_4_^−^ are the dominant species of Cs(I) and Cr(VI) at the optimum pH range, which agrees well with the data reported in the literature (Hamoud et al. 2021; Nasanjargal et al. 2021; Zhang et al. 2021). Natural zeolites have been recognized as excellent adsorbents for cationic species such as Cs^+^ (Belviso et al. 2021; Eljamal et al. 2019; Szala et al. 2015). But, they have little or no affinity for HCrO_4_^−^ where they possess a net negative charge resulting from isomorphic substitution between Si^4+^ and Al^3+^ cations in the crystal lattice (Szala et al. 2015; Haggerty and Bowman 1994). However, organo-modification of natural zeolite with cationic surfactants alters the chemistry of its surface drastically and hence shows strong adsorption for anionic species. This is because cationic surfactants such as cetyltrimethylammonium bromide (C_16_H_33_N(CH_3_)_3_Br), which contains tetrasubstituted ammonium cation with permanently charged nitrogen and a long hydrocarbon chain, are adsorbed onto zeolite via cation-exchange with zeolite’s exchangeable cations with the formation of a monolayer or “hemimicelle” depending on the surfactant concentration. At surfactant concentrations higher than that of its critical micelle concentration (cmc), the hydrophobic groups of the surfactant molecules associate by hydrophobic-hydrophobic attraction to form a bilayer “hemimicelle.” Formation of CTAB “hemimicelle” in the present study, where CTAB concentration of 0.075 mmol/L which is higher than its cmc (Rosen 2004) was used for zeolite modification, at zeolite is suggested to govern through electrostatic attraction at its hydroxyl groups that ionized at high pH values rather than cation-exchange with its exchangeable cations. This suggestion is confirmed by the data obtained by characterization, particularly Figs. 4 and 5 along with the adsorption data achieved at different operating variables (Figs. 6, 9, and 13). Pore size distribution curves and XRD patterns of natural zeolite and CTAB-zeolite clarified that there is no change in pore size and d-spacing, respectively, of zeolite after the modification process. Furthermore, the unchanged maximum adsorption capacity of zeolite for Cs^+^ (Fig. 13) after the modification process again evidenced that the occurrence of CTAB “hemimicelle” at zeolite was not due to cation exchange with the exchangeable cations in its pores. Well-fitting of the kinetic data to the double-exponential kinetic model (Fig. 9) gives rise to an adsorption rate of 3.37 × 10^6^ min^−1^ for HCrO_4_^−^ which is greater than that of Cs^+^ (*K*_3_ = 0.015 min^−1^). These values indicated that HCrO_4_^−^ anions are adsorbed at the external zeolite’s surface while adsorption of Cs^+^ cations is achieved via diffusion into zeolite’s pores. Based on the data given in the present study, it is deduced that adsorption of the studied (radio)toxicants onto CTAB-zeolite are governed by an ion-exchange mechanism where zeolite’s exchangeable cations are replaced by Cs^+^ cations whereas the surfactant’s counter ions (Br^−^) are replaced by HCrO_4_^−^ anions as proposed in Fig. 15.

## Conclusions

Natural zeolite is organically modified using CTAB and evaluated as an adsorbent for simultaneous adsorption of Cs^+^ and HCrO_4_^−^ from aqueous solutions. The occurrence of CTAB at the zeolite surface, which is confirmed by the FTIR, resulted in the formation of smaller particles as evidenced by DLS data. Characterization by nitrogen adsorption–desorption isotherm showed that modification of natural zeolite with CTAB decreased its surface area to nearly a quarter while had no effect on the pore size. The XRD results pointed out that the crystallinity of natural zeolite remained unchanged after the modification process. Results of the effect of solution pH demonstrated that natural zeolite failed to adsorb HCrO_4_^−^ anions efficiently, while CTAB-zeolite succeeded in simultaneously adsorbing Cs^+^ and HCrO_4_^−^ in the pH range 2.5–4.2. The double-exponential kinetic model and the Redlich-Peterson isotherm model are found to be the best kinetic models for fitting the adsorption kinetic and isotherm data, respectively. CTAB-zeolite exhibited maximum adsorption capacities of 0.712 mmol/g (for cesium) and 1.216 mmol/g (for hexavalent chromium). Comparing these values with other studies confirmed the efficiency of the employed adsorbed and its potentiality for the adsorption of cationic and anionic species by a one-step process. Ion exchange is suggested to be the dominant adsorption mechanism in the current study where exchangeable cations of zeolite are replaced by Cs^+^ ions and Br^−^ counter ions of the surfactant are replaced by HCrO_4_^−^.

## Data Availability

All data and materials included in the submitted manuscript will be available upon request.
